# Partial axillary lymph node dissection inferior to the intercostobrachial nerves complements sentinel node biopsy in patients with clinically node-negative breast cancer

**DOI:** 10.1186/s12893-015-0067-4

**Published:** 2015-06-30

**Authors:** Jianyi Li, Shi Jia, Wenhai Zhang, Fang Qiu, Yang Zhang, Xi Gu, Jinqi Xue

**Affiliations:** Department of Breast Surgery, Shengjing Hospital of China Medical University, 36 Sanhao Street, Shenyang, Liaoning Province China

## Abstract

**Background:**

The practice of breast cancer diagnosis and treatment in China varies to that in western developed countries. With the unavailability of radioactive tracer technique for sentinel lymph nodes biopsy (SLNB), using blue dye alone has been the only option in China. Also, the diagnosis of breast malignant tumor in most Chinese centres heavily relies on intraoperative instant frozen histology which is normally followed by sentinel lymph nodes mapping, SLNB and the potential breast and axillary operations in one consecutive session. This practice appears to cause a high false negative rate (FNR) for SLNB. The present study aimed to investigate the impact of the current practice in China on the accuracy of SLNB, and whether partial axillary lymph node dissection (PALND), dissection of lymph nodes inferior to the intercostobrachial nerve (ICBN), was a good complementary procedure following SLNB using blue dye.

**Methods:**

289 patients with clinically node-negative breast cancer were identified and recruited. Tumorectomy, intraoperative instant frozen histological diagnosis, SLNB using methylene blue dye, and PALND or complete axillary node dissection (ALND) were performed in one consecutive operative session. The choice of SLNB only, SLNB followed by PALND or by ALND was based on the pre-determined protocol and preoperative choice by the patient. Clinical parameters were analyzed and survival analysis was performed.

**Results:**

37 % patients with clinically negative nodes were found nodes positive. 59 patients with positive SLN underwent ALND, including 47 patients with up to two positive nodes which were all located inferior to the ICBN. 9 patients had failed SLNB and underwent PALND. Among them, 3 (33.3 %) patients were found to have one metastatic node. 149 patients showed negative SLNB but chose PALND. Among them, 30 (20.1 %), 14 (9.4) and 1 (0.7 %) patients were found to have one, two and three metastatic node(s), respectively. PALND detected 48 (30.4 %) patients who had either failed SLNB or negative SLNB to have additional positive nodes. All the patients with up to two positive nodes had their nodes located inferior to the ICBN. The FNR of SLNB was 43 %. The accuracy rate was 58 %. The follow-up ranged 12–33 months. The incidence of lymphedema for SLNB, PALND, and ALND was 0 %, 0 %, and 25.4 %, respectively (*P* < 0.005). The disease-free survivals for SLNB, PALND, and ALND groups were 95.8 %, 96.8 %, and 94.9 %, respectively (*p* > 0.05).

**Conclusions:**

Under the circumstances of current practice in China, PALND is a good complementary procedure following SLNB in clinically node-negative breast cancer.

## Background

Since the description of the radical mastectomy for patients with breast cancer 100 years ago, axillary lymph node dissection (ALND) has been a necessary part of the operative procedure [1]. Despite the high incidence of arm lymphedema after ALND, before the 1970s most surgeons mainly focused on ensuring patients’ survival rather than minimising this severe complication [2]. In 1980s, advances in chemotherapy, radiotherapy and endocrine therapy significantly improved long term survival [3, 4]. Improving the quality of life for patients with early-stage breast cancer became an important issue. In 1983, Rosen et al. subdivided the axillary lymph nodes (ALNs) into three levels according to their location in relation to the pectoralis minor muscle, and recommended that ALND be carried out from Level I to Level III in a stepwise manner [5]. In the mid-1990s, sentinel lymph node biopsy (SLNB) was introduced [6] and proven to be the best diagnostic modality to accurately stage axilla and select patients with early-stage breast cancer who can be spare of unnecessary ALND and its possible risk of arm lymphedema [7]. The key to SLNB is to accurately identify sentinel lymph nodes (SLNs). Although different methodologies can be used for SLNB, the combined technique of blue dye plus radioactive tracer is significantly superior to blue dye alone technique in terms of negative predictive value and overall accuracy for identification of SLNs [7]. Thus, the combined technique of blue dye with radioactive tracer is generally considered as a gold standard for SLNB.

China is a developing country with considerably uneven economic development and rural–urban inequities in healthcare resources across regions. Compared with western developed countries, the practice of breast cancer diagnosis and treatment in China varies remarkably. With the radioactive tracer technique being applied mainly in research and not being permitted in clinical surgeries, using blue dye alone has been the major technique in large urban medical centres in China where the majority of rural hospitals are unable to provide SLNB service [8]. Meanwhile, with an aim to shorten waiting time and hospital stays to tackle the increasing shortage in healthcare resources, tumorectomy, intraoperative instant frozen histology diagnosis, the potential SLNs mapping and SLNB, and axillary lymph node intervention are normally performed in one consecutive operative session in most medical centres. Low preoperative biopsy rate means that the primary diagnosis of breast cancer relies heavily on intraoperative instant frozen histology. However, the practice of the performing tumorectomy and instant frozen histology prior to SLNs mapping leads to a potentially high false negative rate (FNR) for SLNB because of the effect of open incision on lymphatic drainage. As a result, it has been one of the major goals for the Chinese breast cancer experts to find an easier and more effective method addressing SLN intervention under such circumstances specific to China.

Since 2007, our surgical team has been investigating the metastasis patterns of lymph nodes and distribution of ALNs in relation to the intercostobrachial nerves (ICBNs), which resulted in two publications. We found that ICBN can be an anatomic marker dividing axillary spaces into superior and inferior parts [9]. Short-term follow-up showed that partial axillary lymph node dissection (PALND), dissection of lymph nodes inferior to the ICBNs traced by dye, was a good supplementary procedure to follow SLNB. PALND effectively assessed the status of ALNs and prevented lymphedema after surgery [10]. The long term effects of PALND, however, still remain unknown. The present study aimed to investigate the impact of the current practice in China on the accuracy of SLNB, and whether PALND is a good complementary procedure following SLNB using blue dye. Data from patients with clinically node-negative breast cancer were analyzed to determine the FNR and accuracy rate for SLNB and nodes dissection in relation to ICBN. Survival analysis was also conducted in patients who underwent SLNB only, SLNB followed by PALND or by ALND.

## Methods

### Study population

Between July 2009 and April 2012, a total of 475 patients with suspected malignant breast tumor by preoperative imaging were investigated and approached for consent to participate in the study at the Shengjing Hospital of China Medical University. None of the patients underwent preoperative biopsy. Among them, 289 patients with clinically node-negative breast cancer were identified by intraoperative frozen instant histology and subsequently recruited in this study. Inclusive criteria were: (1) clinically node-negative breast cancer, defined as negative on preoperative axillary palpation, ultra sound examination and CT scan with contrast; (2) no previous history of breast cancer or other malignancies; (3) no neoadjuvant therapy; (4) no pregnancy. Those who had a benign tumor and those who did not meet the above criteria were excluded from the study.

### Study protocols

In the preoperative consultation with the patient and their family, the surgeons provided thorough education and explanation which included the introduction of surgical procedures, study protocols, possible options to choose from if the tumor was proved to be malignant by intraoperative instant frozen histology, significance of SLNB, advantages and disadvantages of blue dye technique and our ongoing study on PALND. The patient was informed that although blue dye alone is considered acceptable for staging and prognostication based on the current guidelines, it may cause false negative result which potentially misses out positive nodes; PALND following SLNB may offset the disadvantage but its long-term complications are unclear. Patients were given enough time to make voluntary decision. An informed consent form and the preference of choice on surgical procedures if the tumor was malignant were obtained. All surgical procedures were performed by the same surgical team. The study was approved by the ethics committee of Shengjing Hospital of China Medical University.

**Fig. 1:**
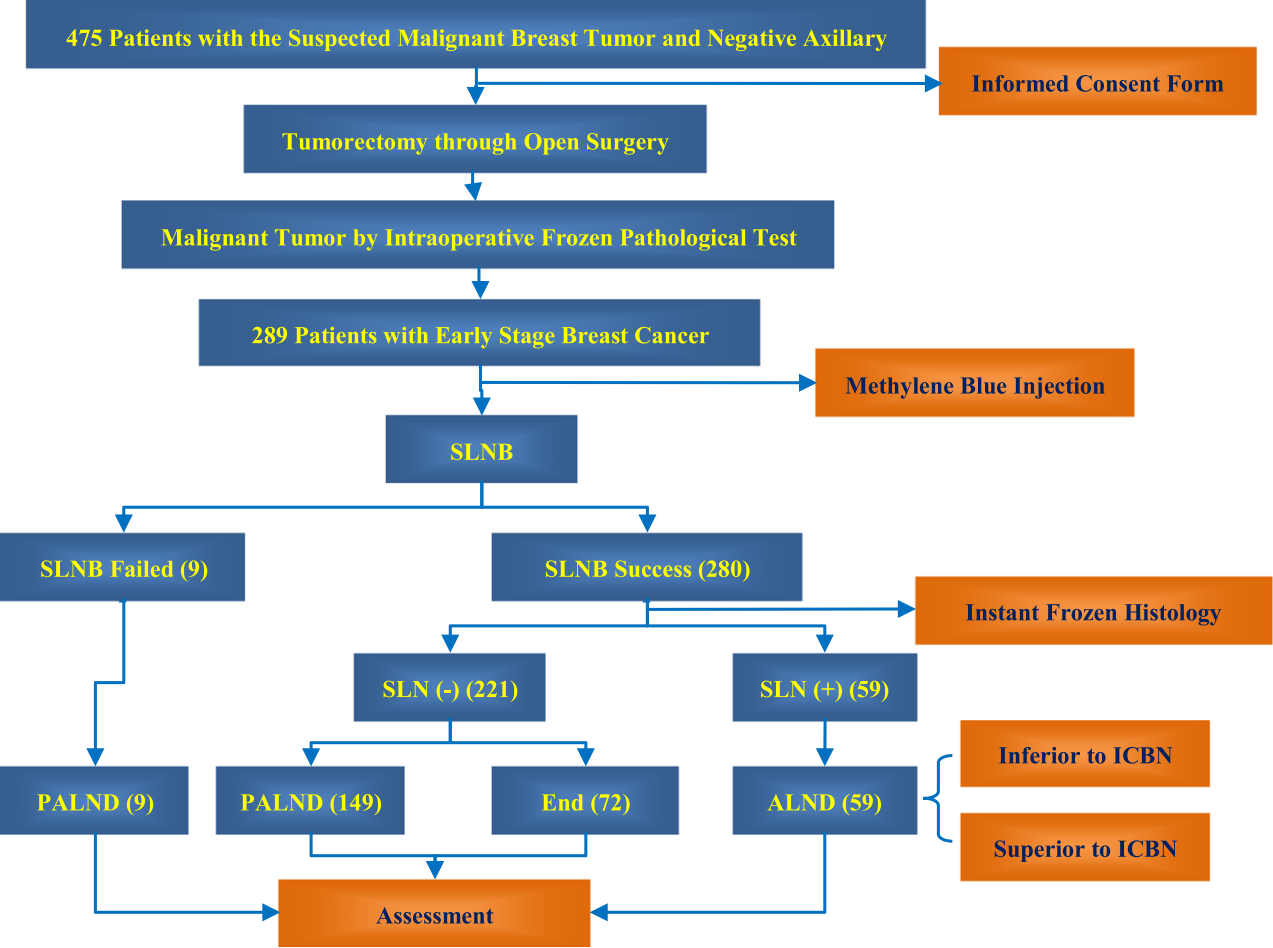
Axillary Intervention in 289 Patients with Early Stage Breast Cancer. Illustration: Firstly, 475 patients with suspected malignant breast tumor by preoperative imaging were investigated and approached for consent to participate in the study at the Shengjing Hospital of China Medical University. Among them, 289 patients with clinically node-negative breast cancer were identified by intraoperative frozen instant histology and subsequently recruited in this study. Then, 289 patients underwent SLNB. 9 patients had failed SLNB who underwent PALND. 280 patients had successful SLNB, among whom 59 patients showed positive SLN and underwent ALND; 221 patients showed negative SLN, 149 of them chose PALND and 72 of them opted for no axillary intervention (end)

All the procedures were done in one consecutive operative session. Firstly, tumorectomy was performed through open surgery and the tumor was examined by intraoperative instant frozen histology analysis. SLN mapping and SLNB were followed if the tumor was proved to be malignant. 2–3 sections were examined for each node investigated by instant frozen histology. If SLNB failed (no blue staining nodes identified), PALND was subsequently performed. If SLNB succeeded and SLN was negative for cancer cells by instant frozen histology, either PALND or no axillary intervention (SLNB only) was followed based on the patient’s preoperative choice. If SLNB succeeded and SLNs showed positive, the patients were offered ALND (Fig. 1).

### Operative techniques

Once the malignant tumor was diagnosed by instant frozen histology, SLNB mapping using methylene blue dye (3–4 ml, 25 mg/ml) was immediately followed as described previously [11]. Briefly, 2/3 of the dye dose was injected into the tumor bed and 1/3 into areola of the breast if the tumor was located in the outer quadrant; if the tumor was located in the inner quadrant, 1/3 of the dose was injected into the tumor bed and 2/3 into areola of the breast. The breast and axillary operations were then performed following 10–15 min massage. We understood that the protocol of performing SLN mapping after tumorectomy would significantly affect the accuracy of SLNB, depending on the location and size of the open incision. However, because this was the common practice in most centres (including ours) in China, one of the aims of the study was to investigate its impact on the accuracy of SLNB.

**Fig. 2:**
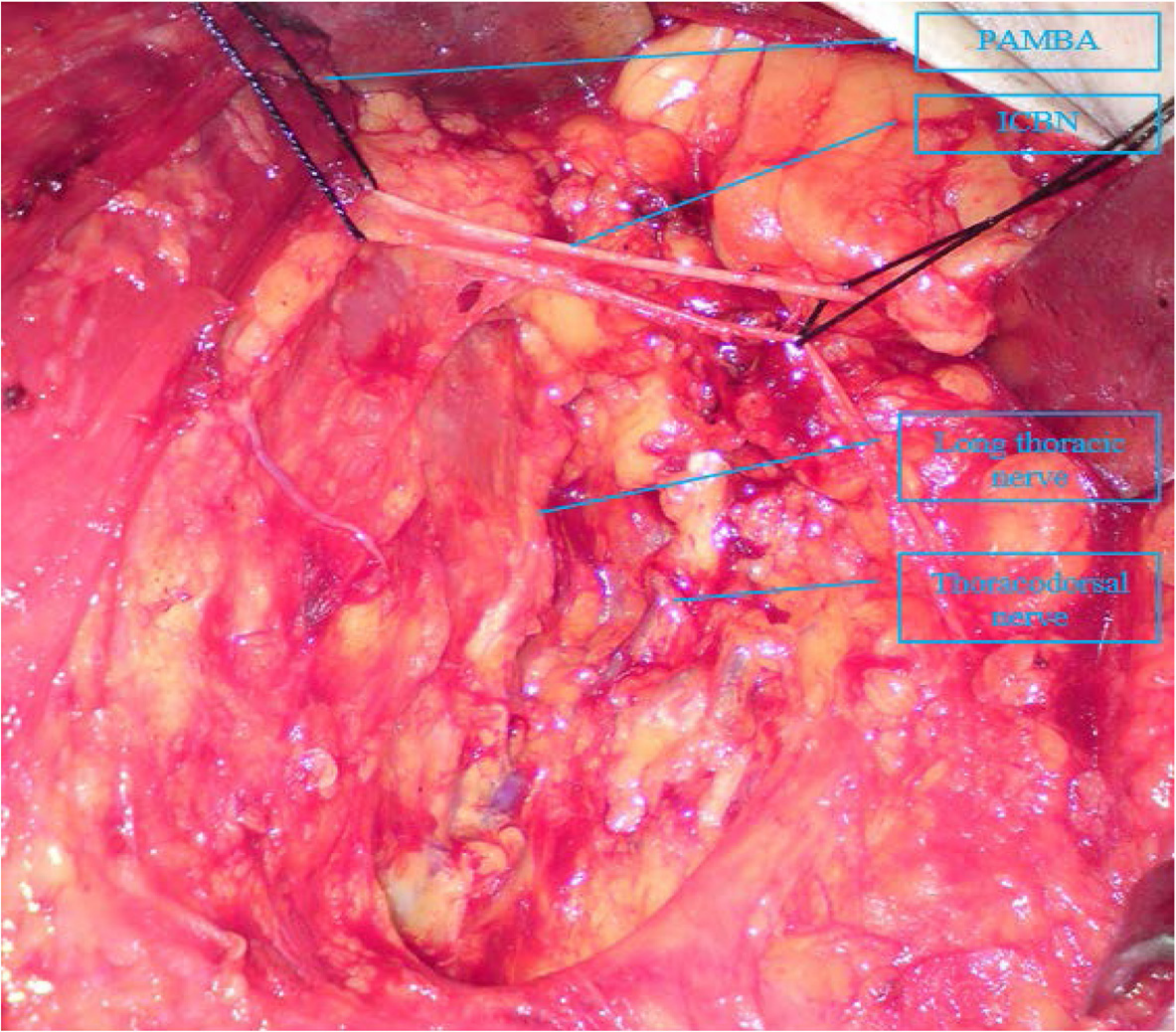
Part of axillary lymph node dissection. Illustration: Part of axillary lymph node dissection (PALND) is bordered by intercostobrachial nerve (ICBN) on the upper margin, by the long thoracic nerve on the median margin, and by the thoracodorsal nerve on the posterior margin. The ICBN is revealed completely in the axillary space, and the lymph nodes adjoining the ICBN belong to the lower lymph nodes

For the axillary operation, the ICBN was exposed completely in the axillary space. All the anatomic locations of soft tissues and lymph nodes removed in relation to ICBN were recorded. The lymph nodes adjoining the ICBN belong to the lower lymph nodes. The long thoracic and thoracodorsal nerves were identified and preserved during the operation [Fig. 2]. The anatomical variations of ICBN were explored during the procedure, and recorded if any was identified. PALND technique involved removal of all the axillary fat pad and lymph nodes inferior to the ICBN. Drainage tube with the same calibre was placed in the axillary space at the end of the operation. All the nodes harvested were postoperatively examined for metastasis by histology.

### Clinical assessment

When the drainage volume was less than 10 ml per 24 h, the tube was removed, and the extubation time and the total volume of drainage were recorded. Body mass index (BMI) and arm circumference at the point of 10 cm proximal to the medial epicondyle were measured before surgery and again one year after surgery. The changes in ipsilateral upper extremity circumference, corrected for any change in the contralateral upper extremity, were calculated using the increase in arm circumference based on the formula introduced by McLaughlin et al. [12]. Severe lymphedema was diagnosed if the increase in arm circumference was greater than or equal to 2 cm during the follow-up period, and mild lymphedema was defined as increase less than 2 cm. We observed substantial weigh gains in many patients after surgery which unexpectedly altered the arm circumference, making this objective tool less accurate. Thus, a subjective method assessing long-term arm lymphedema was proposed and applied: if the patient felt their ipsilateral arms were as good as the contralateral arm, they were defined as no lymphedema; if the patient did not feel that their ipsilateral arms were as good as the contralateral arms but did not affect their daily life, they were defined as mild lymphedema; if their arm lymphedema affect their daily life, they were defined as severe lymphedema.

The collected clinical parameters included: number of nodes, postoperative extubation time, axillary drainage volume, changes in BMI and arm circumference, and other conventional indicators.

### Statistical analysis

All statistical analyses were carried out using SPSS software (version 17.0 for Windows, Chicago, Illinois, USA). The correlation analyses among groups and the various biological factors were examined using the Chi-Square test or analysis of variance (ANOVA). For survival analysis, disease-free survival (DFS) were determined using the Kaplan–Meier curves. The log-rank test was used to compare survival differences among the groups. P value < 0.05 was considered statistically significant.

## Results

### Patient distribution in groups with different intervention

Of the total of 289 recruited patients, no blue-staining SLN was identified in 9 patients who subsequently underwent PALND; therefore the success rate of dye-tracing was 96.9 % (280/289). In 280 patients with successful SLNB, 221 (78.9 %) patients had negative SLN, among whom 149 patients chose PALND and 72 patients opted for no axillary intervention. 59 patients were found SLN positive and underwent ALND as a result. Altogether, 107 out of 289 (37 %) patients with clinically negative nodes were found nodes positive by histology [Fig. 1].

### Characteristics of patients

As shown in Table 1, no differences among groups were found in age, menopause, family history, quadrant distribution, operation type, tumour diameter, pathological types, histological grades, cancer thrombosis, biomarkers (PgR, Ki67 and P53) and targeted therapy (*p* > 0.05). PALND and ALND had higher node metastasis rates (30.4 % and 100 % respectively) compared with the SLNB group (0 %) (*p* < 0.005). The positive Estrogen receptor rates were higher in the SLNB (61.1 %) and PALND (56.3 %) groups than in the ALND group (28.8 %) (*p* < 0.005). The ALND group showed higher positive Her2 rate, higher IIB stage rate, more chemotherapy cycles and higher radiotherapy rate than its two counterparts (*p* < 0.005).

**Table 1:** Characteristics of patients and treatments in all groups

Parameters	SLNB	PALND	ALND	Statistics	*P*
	(*n* = 72)	(*n* = 158)	(*n* = 59)	(F or X^2^)	
**Age**				0.391	0.677
Mean, y	51.68 ± 9.68	52.06 ± 9.90	53.15 ± 10.01		
Range, y	29 ~ 73	25 ~ 82	35 ~ 80		
**Menopause**				1.305	0.521
Yes	37 (51.4 %)	73 (46.2 %)	32 (54.2 %)		
No	35 (48.6 %)	85 (53.8 %)	27 (45.8 %)		
**Family History**				1.412	0.494
No	61 (84.7 %)	142 (90.0 %)	53 (89.8 %)		
Cancer (Including BC)	11 (15.3 %)	16 (10.0 %)	6 (10.2 %)		
Quadrant				10.416	0.237
Areolar	5 (6.9 %)	18 (11.4 %)	8 (13.6 %)		
Outer upper	45 (62.5 %)	83 (52.5 %)	26 (44.1 %)		
Outer lower	17 (23.6 %)	32 (20.3 %)	12 (20.3 %)		
Inner lower	3 (4.2 %)	13 (8.2 %)	9 (15.2 %)		
Inner upper	2 (2.8 %)	12 (7.6 %)	4 (6.8 %)		
**Operation**				6.913	0.141
Mastectomy	54 (75.0 %)	129 (81.6 %)	42 (71.2 %)		
Tumorectomy	13 (18.1 %)	27 (17.1 %)	14 (23.7 %)		
Breast Reconstruction	5 (6.9 %)	2 (1.3 %)	3 (5.1 %)		
**Diameter**	2.61 ± 1.04	2.50 ± 1.00	2.67 ± 1.00	0.686	0.504
**Pathological Types**				12.749	0.546
IDC	47 (65.3 %)	96 (60.8 %)	40 (67.8 %)		
ILC	4 (5.5 %)	13 (8.2 %)	2 (3.4 %)		
DCIS	9 (12.5 %)	24 (15.2 %)	5 (8.5 %)		
Other Types	12 (16.7 %)	25 (15.8 %)	12 (20.3 %)		
**Histological Grade**				7.125	0.129
I	15 (20.8 %)	43 (27.2 %)	23 (39.0 %)		
II	36 (50.0 %)	64 (40.5 %)	24 (40.7 %)		
III	21 (29.2 %)	51 (32.3 %)	12 (20.3 %)		
**Node Metastasis**				145.677	0.000
No	72 (100.0 %)	110 (69.6 %)	0 (0.0 %)		
Yes	0 (0.0 %)	48 (30.4 %)	59 (100.0 %)		
**Cancer Thrombosis**				3.034	0.219
No	47 (65.3 %)	88 (55.7 %)	30 (50.8 %)		
Yes	25 (34.7 %)	70 (44.3 %)	29 (49.2 %)		
**Estrogen Receptor**				16.285	0.000
Negative	28 (38.9 %)	69 (43.7 %)	42 (71.2 %)		
Positive	44 (61.1 %)	89 (56.3 %)	17 (28.8 %)		
**Progesterone Receptor**				5.770	0.056
Negative	40 (55.6 %)	88 (55.7 %)	43 (72.9 %)		
Positive	32 (44.4 %)	70 (44.3 %)	16 (27.1 %)		
**Her2**				17.787	0.000
Negative	62 (86.1 %)	137 (86.7 %)	37 (62.7 %)		
Positive	10 (13.9 %)	21 (13.3 %)	22 (37.3 %)		
**Ki67**				0.010	0.995
Negative	53 (73.6 %)	116 (73.4 %)	43 (72.9 %)		
Positive	19 (26.4 %)	42 (26.6 %)	16 (27.1 %)		
**P53**				1.497	0.473
Negative	41 (56.9 %)	77 (48.7 %)	32 (54.2 %)		
Positive	31 (43.1 %)	81 (51.3 %)	27 (45.8 %)		
**Clinical Stage**				92.884	0.000
0	9 (12.5 %)	14 (8.9 %)	0 (0.0 %)		
I	22 (30.6 %)	49 (31.0 %)	0 (0.0 %)		
IIA	41 (56.9 %)	51 (32.3 %)	16 (27.1 %)		
IIB	0 (0.0 %)	44 (27.8 %)	43 (72.9 %)		
**Clinicopathological Subtypes**				32.593	0.000
Luminal A	37 (51.4 %)	76 (48.1 %)	16 (27.1 %)		
Luminal B Ki67+	13 (18.1 %)	27 (17.1 %)	7 (11.9 %)		
Luminal B Her2+	6 (8.3 %)	9 (5.7 %)	3 (5.1 %)		
Her2 Over-Expression	4 (5.5 %)	12 (7.6 %)	19 (32.2 %)		
TNBC	12 (16.7 %)	34 (21.5 %)	14 (23.7 %)		
**Chemotherapy Protocols**				84.222	0.000
No	6 (8.3 %)	15 (9.5 %)	5 (8.5 %)		
CMF	1 (1.4 %)	8 (5.1 %)	0 (0.0 %)		
CAF or AC	20 (27.8 %)	26 (16.5 %)	0 (0.0 %)		
CEF or EC	18 (25.0 %)	36 (22.8 %)	0 (0.0 %)		
TC or TP	23 (31.9 %)	37 (23.3 %)	14 (23.7 %)		
TAC or AC-T	4 (5.6 %)	36 (22.8 %)	40 (67.8 %)		
**Chemotherapy Cycle**				72.874	0.000
0	6 (8.3 %)	15 (9.5 %)	5 (8.5 %)		
4	19 (26.4 %)	26 (16.5 %)	0 (0.0 %)		
6	43 (59.7 %)	81 (51.2 %)	14 (23.7 %)		
8	4 (5.6 %)	36 (22.8 %)	40 (67.8 %)		
**Targeted Therapy**				4.201	0.122
No	71 (98.6 %)	152 (96.2 %)	54 (91.5 %)		
Yes	1 (1.4 %)	6 (3.8 %)	5 (8.5 %)		
**Radiotherapy**				26.425	0.000
No	54 (75.0 %)	100 (63.3 %)	19 (32.2 %)		
Yes	18 (25.0 %)	58 (36.7 %)	40 (67.8 %)		
**Endocrine Therapy**				20.478	0.002
No	16 (22.1 %)	46 (29.1 %)	33 (55.9 %)		
TAM	22 (30.6 %)	49 (31.0 %)	9 (15.3 %)		
LHRH	12 (16.7 %)	17 (10.8 %)	5 (8.5 %)		
AI	22 (30.6 %)	46 (29.1 %)	12 (20.3 %)		

*SLNB* sentinel lymph node biopsy, *PALND* partial axillary lymph node dissection, *ALND* axillary lymph node dissection, *BC* breast cancer, *IDC* invasive ductal carcinoma, *ILC* invasive lobular carcinoma, *DCIS* ductal carcinoma in situ, *TNBC* triple negative breast cancer, *CMF* cyclophosphamide + methotrexate + 5fluorouracil, *CAF* cyclophosphamide + doxorubicin + 5fluorouracil, *AC* doxorubicin + cyclophosphamide, *CEF* cyclophosphamide + epirubicin + 5fluorouracil, *EC* epirubicin + cyclophosphamide, *TC* docetaxel + cyclophosphamide, *TP* docetaxel + cisplatin, *TAC* docetaxel + doxorubicin + cyclophosphamide, *AC-T* doxorubicin + cyclophosphamide follow docetaxel, *TAM* tamoxifen, *LHRH* luteinising-hormone-releasing hormone, *AI* aromatase inhibitors. All nodes would be histopathological diagnosed to detect macrometastasis

### Clinical outcomes

The number of removed axillary lymph nodes in the ALND group (22.83 ± 5.61) was greater than in the SLNB (3.42 ± 1.45) and PALND (13.53 ± 2.29) groups (*p* < 0.005). Also, the ALND group presented longer postoperative extubation time, greater total drainage volume, and greater increase in BMI and arm circumference compared with its two counterparts (*p* < 0.005) (Table 2). Severe lymphedema evaluated using the absolute changes in arm circumference was not found in the SLNB and PALND groups, 25.4 % severe lymphedema was noted in the ALND group (*p* < 0.005). When using the subjective criteria, severe lymphedema was not reported by any patients in the SLNB and PALND groups, but reported by 30.5 % of patients in the ALND group (*p* < 0.005) (Table 2).

**Table 2:** Clinical outcomes in groups with different axillary intervention

Parameters	SLNB	PALND	ALND	Statistics	*P*
	(*n* = 72)	(*n* = 158)	(*n* = 59)	(F or X^2^)	
Number of LN	3.42 ± 1.45	13.53 ± 2.29	22.83 ± 5.61	631.709	0.000
Postoperative Extubation time (day)	3.49 ± 1.09	4.87 ± 1.43	10.19 ± 2.73	280.783	0.000
Drainage Volume (ml)	284.51 ± 73.37	492.80 ± 75.51	784.75 ± 147.38	458.521	0.000
BMI Preoperative	23.63 ± 3.49	23.48 ± 3.16	23.83 ± 3.69	0.248	0.781
BMI Postoperative	23.85 ± 3.41	23.72 ± 3.16	24.69 ± 3.68	1.882	0.154
△BMI	0.20 ± 0.27	0.24 ± 0.15	0.86 ± 0.63	85.318	0.000
Circumference Preoperative(cm)	24.81 ± 2.61	24.84 ± 2.62	24.84 ± 3.05	0.004	0.996
Circumference Postoperative(cm)	25.05 ± 2.56	25.36 ± 2.60	26.29 ± 3.19	3.682	0.026
△Circumference(cm)	0.26 ± 0.43	0.52 ± 0.30	1.45 ± 0.77	118.540	0.000
Lymphedema by Circumference				118.551	0.000
No	64(88.9 %)	77(48.7 %)	8(13.6 %)		
Mild (<2 cm)	8(11.1 %)	81(51.3 %)	36(61.0 %)		
Severe (△ ≥ 2 cm)	0(0 %)	0(0 %)	15(25.4 %)		
Subjective Lymphedema				105.562	0.000
No	70(97.2 %)	152(96.2 %)	28(47.5 %)		
Mild	2(2.8 %)	6(3.8 %)	13(22.0 %)		
Severe	0(0 %)	0(0 %)	18(30.5 %)		

*SLNB* sentinel lymph node biopsy, *PALND* partial axillary lymph node dissection, *ALND* axillary lymph node dissection

### Anatomic location of blue staining SLNs

**Fig. 3:**
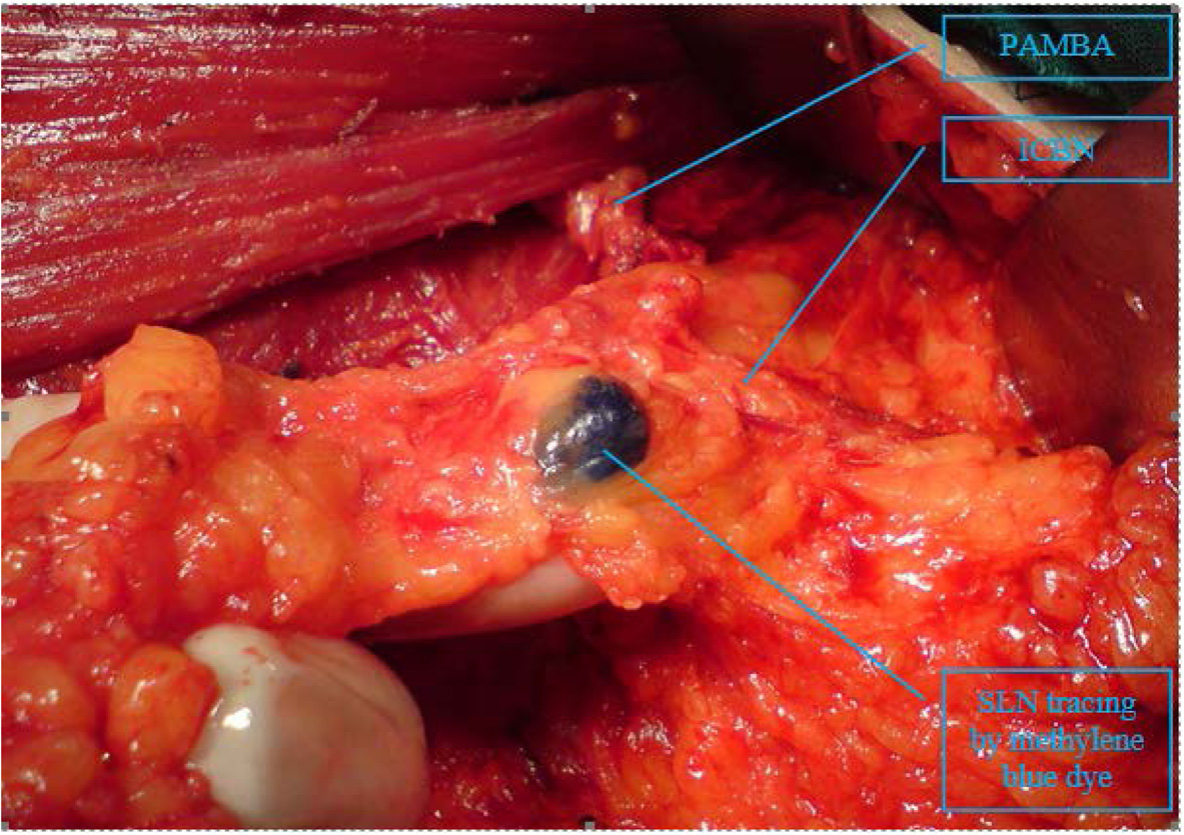
Sentinel lymph nodes located inferior to the intercostobrachial nerve. Illustration: SLN mostly located under the intercostalbrachial nerve (ICBN)

All the methylene blue staining SLNs were found around or inferior to the ICBN in 286/289 (99 %) patients [Fig. 3]. For the anatomical variations of ICBN, we only found one case of ICBN defect. According to a previous study, the pectoralis minor branch of thoracoacromial artery (PMBTA) can substitute ICBN as anatomic landmark [8].

### Distribution of metastatic nodes in patients underwent ALND

As mentioned, 59 patients with positive SLN underwent ALND. Among them, there were 56 patients whose metastatic axillary nodes were all located inferior to the ICBN, including all the patients with one or two metastatic nodes (*n* = 47) and 9 patients with three metastatic nodes. Three patients were found to have metastatic nodes both inferior and superior the ICBN (2 patients with three metastatic nodes and 1 patient with four metastatic nodes) (Table 3).

**Table 3:** Anatomic location of metastatic nodes in patients underwent ALND

Number of LNM	Under the ICBN	Above the ICBN	Patients
1	+	-	16
2	+	-	31
3	+	-	9
	+	+	2
4	+	+	1

*LNM* lymph node metastases, *ICBN* intercostobrachial nerve

### Distribution of metastatic nodes in patients underwent PALND

As stated, 9 patients had failed SLNB and underwent PALND. Among them, 6 (66.7 %) patients were found no metastatic nodes and 3 (33.3 %) patients were found to have one metastatic node postoperatively. Meanwhile, 149 patients showed negative SLNB but chose PALND. Among them, 104 (69.8 %) patients were proved node metastasis free. However, 30 (20.1 %), 14 (9.4) and 1 (0.7 %) patients were found to have one, two and three metastatic node(s), respectively (Table 4). Putting together, PALND detected 48 (30.4 %) out of 158 patients who had either failed SLNB or negative SLNB to have additional positive axillary nodes.

**Table 4:** Distribution of metastatic nodes in patients of all groups

Number of LNM	SLNB failed	SLNB negative		SLNB positive	X^2^	*P*
	PALND after SLNB(9)	PALND after SLNB(149)	End after SLNB(72)	ALND after SLNB (59)		
0	6 (66.7 %)	104 (69.8 %)	-	0	181.568	0.000
1	3 (33.3 %)	30 (20.1 %)	-	16 (27.1 %)		
2	0	14 (9.4 %)	-	31 (52.5 %)		
3	0	1 (0.7 %)	-	11 (18.6 %)		
4	0	0	-	1 (1.7 %)		

LNM = lymph node metastases. False negative rate (FNR) of SLNB using blue dye: 45 % (48/107), accuracy: 55 %

### FNR of SLNB

If the nine patients with failed SLNB were not included in the analysis, the FNR of SLNB using blue dye in the present study was 43 % (45/104). The accuracy rate was 58 % (163/280) (Table 4).

### Outcomes of follow-up

**Fig. 4:**
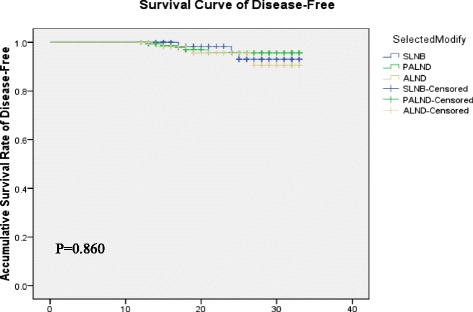
Kaplan-Meier Curves for Disease-free Survival

The follow-up time ranged from 12 to 33 months in each group with median 24 months, 20.5 months and 22 months for SLNB, PALND and ALND, respectively (*p* > 0.05). The actual DFS of SLNB, PALND, and ALND groups were 95.8 %, 96.8 %, and 94.9 %, respectively (*p* > 0.05), and the median DFS time was 30 months in each group [Table 5]. No differences were observed among groups in the curves for DFS [Fig. 4]. There was no difference in local recurrence among groups.

**Table 5:** Survival analysis according to axillary intervention

Parameters	SLNB	PALND	ALND	Statistics	*P*
	(*n* = 72)	(*n* = 158)	(*n* = 59)	(F or X^2^)	
Overall Survival	97.2 %	98.1 %	96.6 %	0.445	0.796
Deaths (n)	2	3	2		
Median Survival Time (mo)	30.0	30.0	30.0	0.251	0.882
Disease-Free Survival	95.8 %	96.8 %	94.9 %	9.033	0.340
Event (n)	3	5	3		
Local Recurrence	0	0	0		
Contralateral breast cancer	0	2	0		
Bone Metastasis	1	0	0		
Lung Metastasis	0	0	1		
Multi-Organ	2	3	2		
Disease-Free Survival (mo)	30.0	30.0	30.0	0.303	0.860
Follow-Up Time				1.633	0.197
Median (mo)	24.0	20.5	22.0		
Range (mo)	12-33	12-33	12-33		

*SLNB* sentinel lymph node biopsy, *PALND* partial axillary lymph node dissection, *ALND* axillary lymph node dissection, *mo* months

## Discussion

As preservation of the ICBNs does not affect patient survival but significantly ameliorates the sensory deficit and other long-term symptoms [13], this procedure potentially provides a new anatomic landmark in axillary lymph nodes dissection. In our previous study investigating the anatomic localization of SLNs in relation to the ICBN, we found that the majority of SLNs were located inferior to the ICBN using a dye-tracer technique, and axillary lymph nodes metastases progress from the inferior to the superior axillary as divided by ICBN [9]. This is similar to the findings by Clough et al. who discovered that nearly 89 % SLNs were inferior to the ICBN and few were located superior to ICBN and at the outside of the subscapular artery when traced by the combined method [14]. A recent study demonstrated that the node draining the arm lymph vessels was localized in the lateral pillar of the axillary, and was always found superior to or at the level of the second ICBN [15]. These findings indicate that the localization of nodes draining the breast and the arm lymphatic vessels is not overlapping and is largely divided by ICBN. Theoretically, postoperative arm lymphedema is preventable if the tissue and nodes superior to ICBN are preserved. PALND, which only involves dissection of nodes inferior to ICBN, should have similar lymphedema occurrence rate with SLNB but lower rate than ALND. In the present study, no postoperative severe arm lymphedema was found after one year follow-up in the SLNB and PALND groups with over 15 % of severe lymphedema in the ALND group, suggesting PALND has the same protective effect as SLNB on arm lymphedema and is superior to ALND because the lymphatic drainage of the arm was preserved. The high level of chemotherapy, later clinical stage, and the poorer clinicopathological subtypes in the ALND group may have also contributed to the lymphedema observed in this group.

We carried out survival analysis according to the types of axillary intervention. There was no difference among the SLNB, PALND and ALND groups in DFS with a zero recurrence rate in all groups. Patients in the ALND group might have benefited from chemotherapy and radiotherapy. Our findings are consistent with a study by Kodama et al. who compared overall 5-year survival rates in patients undergoing PALND and historical controls undergoing conventional dissection [16]. Thus, there is sufficient evidence to suggest that PALND can significantly prevent arm lymphedema with a satisfying DFS.

Blue dye is so far the first choice for SLNB in China because it is cheap, safe without the need for nuclear medicine department and gamma probes. However, although blue dye alone technique is generally considered as a valid and acceptable tool to assess axillary nodes status and staging [17], there is growing evidence suggesting the combined technique of blue dye plus radioactive tracer is more superior. In a randomized study by Radovanovic et al., the false rate of blue dye was 17.6 % and accuracy was 68 % [7]. Other studies in the literature showed the similar false-negative rates for the blue dye alone method which were generally higher than the recommended 5 % rate [18, 19]. The combined blue dye and radioactive tracer had a significantly lower FNR (4.5 %) with higher accuracy [7]. In the present study, the FNR of blue dye technique was as high as 43 % and the accuracy rate was only 58 %, which appeared to be unexpected and unacceptable. We believe that the practice of performing SLN mapping/SLNB after tumorectoy is largely responsible for the high FNR and low accuracy rate as open incision and the tumor removal procedure are potentially destroy breast lymphatic network, thus reduce breast lymphatic drainage towards axilla. The lack of other intensive node exploration technique (e.g. continuous multiple sections and lymph node imprint) in the present study may have also been a contributor. Different from western countries where malignance of a tumor is determined by preoperative biopsy and surgical plan is normally determined preoperatively, such practice is common in China where malignance of a tumor is largely identified by intraoperative instant frozen histology and the therapeutic plan is determined intraoperatively. With unknown biological behaviour of a tumor and uncertain time frame before the result of intraoperative frozen histology was available, it appeared inappropriate to perform SLNB prior to tumorectomy. Our study showed an unacceptably higher FNR using blue dye alone under such circumstances.

We found that 48 (30.4 %) patients who showed either failed SLNB (33.3 %) or negative SLNB (30.2 %) using blue dye were found additional positive nodes by PALND. These data demonstrated that there may be an over 30 % chance of leaving behind positive nodes for those with failed or negative SLNB using blue dye alone. In a country where blue dye is the only option for SLNB and intraoperative instant frozen histology is the major method for primary diagnosis of breast cancer, PALND can be considered as a good complementary procedure to SLNB in patients with clinically-negative node breast cancer. Our findings are in keeping with an Indian study which showed low axillary sampling is accurate in predicting axillary nodes status in women with clinically node negative operable breast cancer [20]. In the light of the high FNR of SLNB, we are now trialling SLNB prior to tumorectomy to minimise the effect of open incision on breast lymphatic drainage in all patients with suspected breast cancer. The FNR and accuracy rate are yet to be evaluated.

Recently, a presentation and related publications of the American College of Surgeons Oncology Group (ACOSOG) Z0011 have provoked controversy around the world regarding the need for ALND in patients with SLN-positive breast cancer [21–23]. It seems that less than or equal to two positive nodes does not warrant dissection. In our study, all the patients with less than or equal to two positive SLNs had all their metastatic nodes located inferior to the ICBN. Only three patients with more than two metastatic nodes had nodes found superior to the ICBNs. Therefore, PALND is considered as a way of biopsy for clinically node-negative breast cancer: if there are two or fewer positive nodes, then ALND is not necessary and the PALND is sufficient; if there are more than two positive nodes, then ALND is necessary. A recent study reported that for patients with positive nodes who do not undergo ALND, adjuvant therapy can have the same effect on overall survival as the ALND procedure performed on similar node-positive patients [24]. However, the presence of positive nodes is one of the important factors to be taken into account when considering radiotherapy and chemotherapy. Perhaps in the near future we can develop a novel imaging technique instead of SLNB for the assessment of ALNs, we will enter a new era when the axillary surgery is not necessary. However, at the present time, PALND could be considered as a procedure complementary to SLNB in clinically node-negative breast cancer, especially in China where the combined technique is not available for SALN detection. This technique is so simple that a surgeon could easily perform without the need of complicated equipment. In fact, we prefer the name “functional axillary lymph node dissection (FALND) to PALND, a term which is coined when SLNs are not located. Our further investigation is ongoing on the clinical difference between FALND and ALND when there are two or fewer positive nodes and the results will be available in the near future.

In conclusion, there are considerable differences in the practice of breast cancer diagnosis and treatment between China and western developed countries. In a country where blue dye is the only technique for SLNB and intraoperative SLNB is normally performed after tumorectomy, the FNR of SLNB is unacceptably high. Under such circumstances specific to China, axillary node dissection inferior to the ICBN is a good complementary procedure following SLNB in clinically node-negative breast cancer because it provides better assessment of nodes status, reduces the occurrence rate for postoperative arm lymphedema with satisfying long-term disease free survival.

## Conclusions

The current practice in China where SLNB is generally performed after tumorectomy and intraoperative instant frozen histology reduces the accuracy of SLNB using blue dye. Under such specific circumstances, PALND is a good complementary procedure following SLNB in clinically node-negative breast cancer.
